# T2-imaging of area-at-risk predicts recovery of cardiac function in a canine model of acute myocardial infarction

**DOI:** 10.1186/1532-429X-11-S1-P43

**Published:** 2009-01-28

**Authors:** Jamieson M Bourque, David K Glover, Craig C Goodman, Mirta R Herrera, Fred Epstein, George A Beller, Christopher M Kramer

**Affiliations:** University of Virginia, Charlottesville, VA USA

**Keywords:** Acute Myocardial Infarction, Infarct Size, Canine Model, Cine Imaging, Myocardial Edema

## Introduction

Infarct size is the strongest predictor of subsequent outcomes and remains an important therapeutic target. Myocardial edema measured by T2-weighted magnetic resonance (MR) imaging identifies the ischemic area-at-risk and predicts infarct size, allows more accurate evaluation of novel anti-ischemic therapies, and may be associated with stunning and long-term recovery of function.

## Purpose

We sought to identify the extent of infarcted and at-risk myocardium and correlate these markers with post-infarct recovery of myocardial function.

## Methods

Five dogs (mean weight 18.9 kgs) underwent successful 2-hour complete occlusion of the left-circumflex artery and collaterals after baseline functional cine imaging. They subsequently underwent 48-hour and 4-week complete left-ventricular (LV) short-axis MR imaging on a 1.5 Tesla scanner using SSFP-cine (TE 1.4 ms, TR 35.3 ms, voxel size 0.6 × 1 × 7 mm^3^), ACUTE T2-weighted (TE 1.9 ms, TR 168.1 ms, voxel size 0.6 × 0.9 × 7 mm^3^), and phase-sensitive, inversion recovery late Gadolinium (0.15 mmol/kg Magnevist) enhancement sequences (LGE, TE 3.4 ms, TR 548 ms, voxel size 0.5 × 0.8 × 7 mm^3^). The percentages of LGE and T2 hyperenhacement were identified through intensity threshold testing, 5 standard devitations (SDs) and 2 SDs above the reference myocardium respectively. All outcome variables were given as mean percentages ± SDs.

## Results

**Figure 1:**
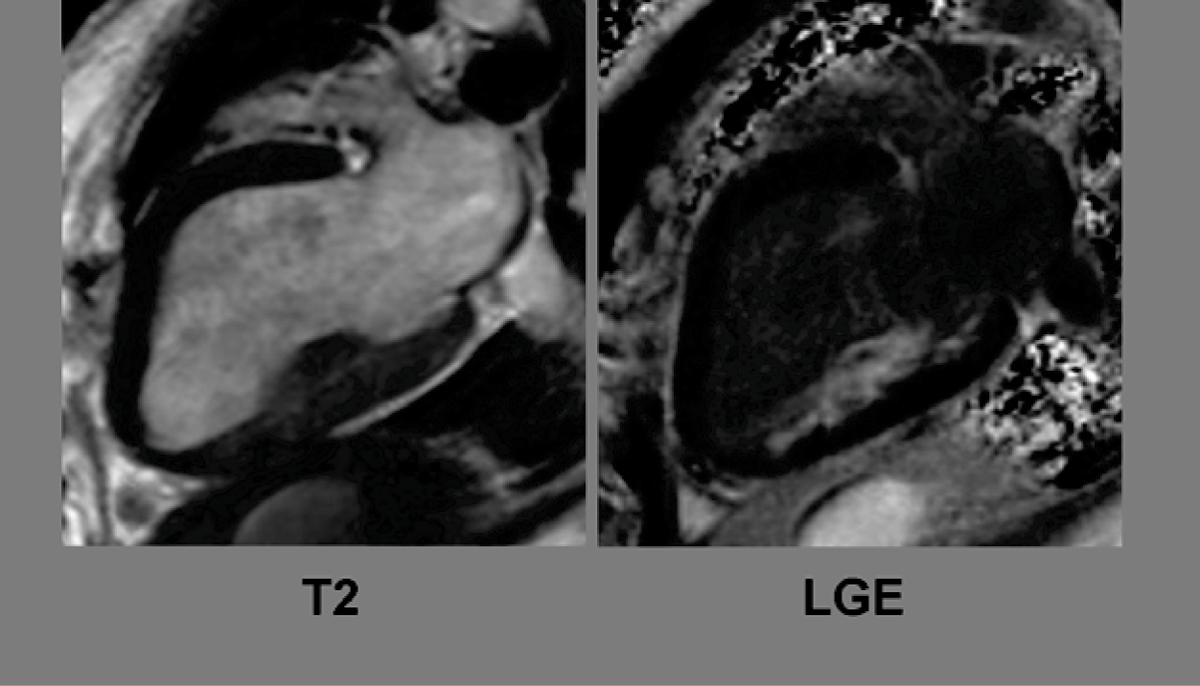
Figure 1

The mean percentage LGE was extensive at 44.1 ± 5.9%. T2-imaging identified a large area-at-risk measuring 68.5 ± 7.1% of the myocardium. The mean LV ejection fraction (LVEF) at 48 hours was 23.7 ± 2.8%. Significant recovery of function was seen at four weeks (mean LVEF increased to 42.7 ± 7.3%), likely in large part due to the significant stunning present and low LGE/T2 hyperenhancement ratio (0.65 ± 0.13) at 48 hours. This ratio was found to moderately correlate negatively with the percent LVEF improvement (r = -0.57). Figure 1.

## Conclusion

The area-at-risk estimated by T2-weighted MR imaging correlates with recovery of myocardial infarction in an acute-MI canine model. Further evaluation of this method will maximize our ability to prognosticate and guide therapy post-infarction. Increasing the proportion of myocardial salvage from the initial area-at-risk should serve as an important therapeutic target. This imaging technique may become a significant marker of treatment efficacy and prognosis.

